# Topical streptomycin irrigation of lesions to prevent postoperative site infections in spinal tuberculosis: a retrospective analysis

**DOI:** 10.1186/s13018-023-04059-y

**Published:** 2023-08-10

**Authors:** Jianqiang Du, Wenxiu Qin, Yanjun Zhang, Zhengyuan Yang, Junjie Li, Jun Yang, Qiang Deng

**Affiliations:** 1 Gansu University of Chinese Medicine, Lanzhou, China; 2 First Teaching Hospital of Tianjin University of Traditional Chinese Medicine, Tianjin, China; 3 Department of Orthopaedic Spine II, Gansu Provincial Hospital of Traditional Chinese Medicine, 418 Guazhou Road, Qilihe District, Lanzhou City, Gansu Province China

**Keywords:** Tuberculosis of the spine, Surgery, Streptomycin, Local irrigation of the lesion, Surgical site infection

## Abstract

**Purpose:**

In spinal tuberculosis surgery, topical administration of drugs to the lesion is a preventive treatment measure. The aim is to achieve better bacterial inhibition and to prevent complications. As one of the most common complications after spinal tuberculosis surgery, many factors can lead to surgical site infection (SSI). No definitive reports of local streptomycin irrigation of the lesion and SSI of spinal tuberculosis have been seen. This study analyzed data related to surgical site infections (SSI) after the treatment of spinal tuberculosis using this regimen.

**Methods:**

In this study, 31 were in the observation group (streptomycin flush) and 34 in the control group (no streptomycin flush). All patients received the same standard of perioperative care procedures. General information, operative time, intraoperative bleeding, ESR and CRP at one week postoperatively, time on antibiotics, total drainage, days in hospital, incision infection rate and secondary debridement rate were compared between the two groups.

**Results:**

Patients in both groups completed the surgery successfully. The ESR and CRP levels in the observation group were lower than those in the control group one week after surgery (*p* < 0.05); the duration of postoperative antibiotics and hospital stay were lower than those in the control group (*p* < 0.05); the incidence of SSI in the two groups was 5.88% and 6.45% respectively, with no significant difference (*p* > 0.05).

**Conclusion:**

The use of topical streptomycin irrigation of the lesion during surgical procedures for spinal tuberculosis had no significant effect on the incidence of SSI, however, it helped to control the level of infection in the postoperative period and reduced the length of time patients had to use postoperative antibiotics and the number of days they stayed in hospital. Future prospective randomised controlled trials in more centres and larger samples are recommended.

## Introduction

Tuberculosis (TB) is one of the oldest known human infectious diseases and an estimated 2 billion people worldwide are infected with TB [1]. By 2021, there will be 10.6 million new cases of tuberculosis and 1.6 million deaths from the disease worldwide [2]. At the same time, the number of people with drug-resistant TB continues to grow. The tuberculosis epidemic remains serious worldwide, and the high burden of tuberculosis is even more acute in developing countries. Spinal tuberculosis (ST) is one of the extrapulmonary forms of tuberculosis caused by Mycobacterium tuberculosis attacking the spine. Of the entire spine, TB occurs most frequently in the lumbar spine, followed by the thoracolumbar segment, and less frequently in the cervical and lumbosacral regions. The exact incidence of spinal TB is not known, but skeletal TB is found in nearly 10% of patients with active pulmonary disease [3].The spine is the most common site of skeletal involvement, accounting for 50%-75% of bone and joint tuberculosis [4]. The incidence of tuberculosis of the spine is currently increasing year by year and can occur in all age groups [5].

Tuberculosis of the spine is slow in onset and early symptoms are insidious. Systemic symptoms often include low-grade fever, night sweats, weakness, loss of appetite and anaemia. Local symptoms often include pain, restricted spinal movement and neurological dysfunction. Pain is the first to appear, and as the disease progresses, symptoms of paraplegia and kyphosis may appear in later stages [6].The first step in treating patients diagnosed with spinal tuberculosis is early, combined, regular, moderate, and complete treatment with anti-tuberculosis drugs. If the disease progresses to an advanced stage, leading to kyphosis, neurological deformity or even paraplegia, active surgical intervention is an effective adjunct to the treatment of spinal tuberculosis. Although surgery offers good results for patients with spinal TB, surgical site infection (SSI) is one of the most common post-operative complications of spinal TB. Patients with spinal TB suffer from chronic malnutrition and immune deficiencies, and tend to have a higher incidence of SSI after surgery compared to patients with general spinal surgery [7]. Once SSI occurs, it affects not only the healing of the surgical incision but also increases the patient's pain and financial burden. Therefore, as the number of spinal TB procedures increases, controlling SSI is also one of the key challenges facing surgeons.

Streptomycin is a basic aminoglycoside compound that binds to the proteins of the Mycobacterium tuberculosis ribonucleic acid proteome to interfere with Mycobacterium tuberculosis protein synthesis, thereby killing or inhibiting the growth of Mycobacterium tuberculosis. It has therefore remained the first line of anti-tuberculosis drugs for decades. In the early days, 25 cases of post-operative fistulas for osteoarticular tuberculosis were treated with topical streptomycin injections [8], which were found to have satisfactory antibacterial effects. This pioneered the local treatment of osteoarticular tuberculosis in clinical practice. Later clinical practitioners applied this method to spinal tuberculosis surgery to achieve prophylactic treatment results. The use of streptomycin powder has been shown to be effective in reducing postoperative SSI in spinal tuberculosis. However, there may be some shortcomings in the local application of streptomycin powder. This study reports a comparative analysis of the role of topical streptomycin irrigation of lesions in the prevention of SSI in patients undergoing surgical treatment for spinal tuberculosis.

## Information and methods

### Patient population

Retrospective analysis of 65 patients with spinal tuberculosis who underwent surgical treatment in our hospital from January 2019 to January 2022, diagnosed with spinal tuberculosis based on clinical presentation and laboratory (ESR, CRP), imaging (x-ray, CT, MRI) and pathological examination. The inclusion criteria were: (1) diagnosis of spinal tuberculosis and age ≥ 18 years. (2) Significant bone destruction, spinal instability, nerve damage, abscesses, or the presence of dead bone. (3) No history of streptomycin allergy. (4) Complete documentation of clinical data. Exclusion criteria: (1) Patients with active pulmonary tuberculosis or malignancy. (2) Recurrent spinal tuberculosis. (3) Patients with severe heart, lung, liver or kidney disorders. (4) The clinical history was not fully documented. In this study, All patients were operated on using a posterior approach for lesion removal, interbody bone grafting and internal fixation with a nail rod system. The patients were divided into observation and control groups according to the presence or absence of intraoperative topical streptomycin irrigation of the lesion.

### Preoperative preparation

All patients underwent X-ray, CT and MRI to assess the extent of destruction of the affected vertebral body, the distance between the vertebral spaces, the presence of cold abscess formation and the presence of nerve root compression, which helped to make the pre-operative diagnosis and surgical strategy. Patients in both groups were treated preoperatively with conventional antituberculosis therapy for at least 2 weeks in a quadruple chemotherapy regimen consisting of isoniazid + rifampicin + ethambutol + pyrazinamide. Patients with malnutrition (haemoglobin < 10 g/L and albumin < 30 g/L) are given intravenous nutritional support to correct anaemia, electrolyte disturbances and hypoproteinaemia.

### Surgical methods

The procedure is timed from the time the skin is cut to the time the sutures are completed. The patient is placed in the prone position and the airway is intubated with general anaesthesia. A standard dorsal midline approach is used, with a surgical incision along the spinous process centred on the lesion. The subcutaneous tissue is incised and the muscles and fascia on both sides are stripped along the spinous process. The lesion is located under conventional X-ray fluoroscopy and a nail is placed in the vertebral body adjacent to the lesion. The spinous processes at the site of the lesion are cut away with spinous scissors and then the severely diseased lamina and ligamentum flavum are removed with biting forceps, and the articular processes, pedicles and transverse processes on the side of the lesion are removed to ensure a clear surgical view. The spinal cord is protected with a pulling hook to expose the tuberculous lesion. Necrotic tissue, including diseased discs and dead bone, is removed from the lesion. Then, a total of 4.0 g of streptomycin powder is diluted into 2000 ml of 0.9% sterile sodium chloride solution, and the cleaned lesion is fully irrigated locally. At the same time, the waste fluid was pumped out through the negative pressure suction head, which could remove the residual pus and tissue debris and effectively prevent the spread of contamination sources. In the control group, only 2000 ml of 0.9% sterile sodium chloride solution was used for local irrigation of the lesion without streptomycin. A suitable autogenous or allogeneic bone is embedded between the vertebrae. Suitable autogenous or allograft bone is inserted between the vertebrae, the titanium rods are fixed bilaterally, screws are attached and appropriate pressure is applied to secure the implants. X-rays are taken to determine the corrective effect and the position of the bone graft fixation. After these operations are completed, the incision is carefully haemostatic. Finally, a negative pressure drainage tube is placed, sutured layer by layer and the incision is covered with sterile gauze.

### Postoperative management

Post-operative prophylactic antibiotic treatment and monitoring of incisional changes. If leakage from the incision is detected, the duration of postoperative antibiotics may be extended. Remove the drainage tube when the drainage flow is less than 20 ml and the drainage fluid is clear and yellowish in colour 24 h after surgery. 3–5 days after surgery, the patient stands or walks in a brace, which is worn for at least 3 months. Post-operative routine quadruple anti-tuberculosis chemotherapy for 12–18 months. Post-operative monthly follow-up visits to evaluate ESR, CRP levels and liver and kidney function. Full spine x-rays and CT or MRI if necessary are repeated at 1, 3, 6 and 12 months after discharge.

### Intraoperative and postoperative observation indexes

The two groups were compared in terms of operative time, intraoperative blood loss, ESR and CRP levels at one week postoperatively, total postoperative drainage, length of hospital stay, duration of antibiotic use, incidence of SSI and debridement. Patients were followed up for at least 6 months to determine the post-operative outcome of spinal TB.

### Statistical processing

SPSS26.0 statistical software was used for analysis. The measurement data were described by mean ± standard deviation, and independent sample *t*-test was selected for comparison between groups; the count data were described by frequency (percentage), and the chi-square test or Fisher exact probability method was used for comparison between groups. *p* < 0.05 was considered a statistically significant difference.

## Results

A total of 65 subjects were included in this study, 31 cases in the observation group group, 14 males and 17 females, with a mean age of 47.9 ± 15.1 years, of which 34 cases in the control group, 16 males and 18 females, with a mean age of 45.1 ± 15.1 years. In terms of the site of surgery, i.e. the segment where the diseased vertebrae were located, lumbar TB was predominant in both groups (44.12 and 45.16), followed by thoracic (26.47 and 25.81) and thoracolumbar (17.65 and 22.58), and less frequently lumbosacral TB (11.76 and 6.45). The operative time and intraoperative bleeding were essentially similar in the two groups. Overall, there were no statistically significant differences in the comparison of general information between the two groups of patients (all *p* values > 0.05), as shown in Table 1.

**Table 1:** Comparison of general information of patients in the two groups

	Control group	Observation group	*p*
Age	45.1 ± 15.1	47.9 ± 15.1	0.471
Duration of disease	19.2 ± 7.9	17.9 ± 7.4	0.515
Sex			0.878
Male	16 (47.06)	14 (45.16)	
Female	18 (52.94)	17 (54.84)	
Surgical site			0.917
Thoracolumbar spine	6 (17.65)	7 (22.58)	
Thoracic spine	9 (26.47)	8 (25.81)	
Lumbosacral	4 (11.76)	2 (6.45)	
Lumbar spine	15 (44.12)	14 (45.16)	
Operative time Surgical bleeding	204.94 ± 24.00	201.94 ± 27.13	0.637
Surgical site	417.65 ± 127.23	425.48 ± 119.61	0.799

We compared the postoperative infection indicators between the two groups and showed that localized saline irrigation of spinal TB lesions with streptomycin was effective in reducing ESR and CRP levels at 1 week postoperatively (both *p* values < 0.05). In the comparison of incisional drainage, the observation group appeared to be lower than the control group (379.03 ± 69.92 vs. 387.65 ± 68.00), but not statistically significant (*p* > 0.05). The mean length of stay in the observation group was 17.19 ± 2.88 days; lower than the 19.82 ± 4.03 days in the control group (*p* = 0.004). The use of topical streptomycin irrigation was able to reduce the mean postoperative antimicrobial drug use time, which was 5.71 ± 1.60 days in the observation group, lower than the control group (7.82 ± 1.75) (*p* < 0.001). In this study, two SSIs occurred in each of the two groups and the use of topical streptomycin irrigation had no significant effect on SSI in spinal tuberculosis (*p* > 0.05). A total of 4 SSIs occurred in all patients, with a second admission selected, all of which healed after debridement surgery, as shown in Table 2.

**Table 2:** Comparison of infection data between two groups of patients

	Control group	Observation group	*p*
Blood sedimentation one week after surgery	35.98 ± 6.36	30.45 ± 7.52	0.002
CRP at one week postoperatively	24.01 ± 7.87	19.34 ± 5.98	0.01
Total drainage	387.65 ± 68.00	379.03 ± 69.92	0.616
Length of hospitalization	19.82 ± 4.03	17.19 ± 2.88	0.004
Duration of postoperative antibiotic use	7.82 ± 1.75	5.71 ± 1.60	< 0.001
Postoperative infection			1
No	32 (94.12)	29 (93.55)	
Yes	2 (5.88)	2 (6.45)	
Secondary debridement			1
No	32 (94.12)	29 (93.55)	
Yes	2 (5.88)	2 (6.45)	

## Discussion

Although tuberculosis has been effectively prevented and controlled, the incidence of spinal tuberculosis has been on the rise in recent years. On top of anti-tuberculosis treatment, surgical treatment with lesion removal can significantly improve the outcome of spinal tuberculosis treatment. As a wasting disease, TB is often characterised by wasting, low BMI, reduced serum albumin and reduced resistance and immunity. Patients undergoing surgical treatment have an increased need for nutrients such as albumin in response to trauma and surgical site repair. Inadequate nutrition at this time increases the risk of postoperative surgical site infections in patients undergoing tuberculosis spine surgery, despite strict adherence to protocols for preventing surgical site infections. This ultimately leads to internal fixation failure, spinal instability, neurological dysfunction and even sepsis and death, as well as increased medical costs [9].

The incidence of SSI after spinal surgery is approximately 1–14% [10]. Superficial infections at the site of spinal surgery can be cured by the use of sensitive antibiotics and surgical dressing changes. However, deep post-spinal incisional infections and adjacent tissue-organ gap infections are located in a dead space beneath the deep fascia and are usually uniformly classified as deep post-spinal incisional infections [11]. Antibiotics are the basis for the treatment of deep post-operative spinal incisional infections, and identification of the type of infecting bacteria is essential for the selection of antibiotics. Deep post-spinal incisional infections should be treated with broad-spectrum antibiotics as soon as possible after they occur. Particularly in unstable patients such as sepsis, exudate or puncture fluid should also be retained for bacterial culture and further sensitive antibiotics should be selected based on the culture results [12].

In spinal surgery, the role of intra-incisional topical antibiotics has been extensively studied for the effective prevention of surgical site infections. The use of topical antibiotics creates a high concentration of antibiotics in a small area with low blood levels, while avoiding the toxic side effects of systemic administration [13]. Glassman et al. [14] achieved good results in patients with incisional infections after internal spinal fixation by placing antibiotic bone cement in the form of cement chain beads on the lateral and superficial layers of the deep fascia of paravertebral internal fixation in combination with debridement and irrigation. lemans et al. [15] in a meta-analysis evaluated the role of intra-wound antibiotics in spinal surgery in preventing surgical site infections and concluded that deep incisional infections were reduced by 3–7 times in spinal surgery.Dennis et al. [16] compared all 389 patients who underwent spinal surgery at a single institution. The results showed that prophylactic intraoperative topical vancomycin powder reduced the risk and morbidity of SSI in spinal surgery (*p* = 0.049).

Previously, Yang et al. [17] conducted a retrospective analysis of 161 patients with spinal tuberculosis who underwent surgical treatment and found that intraoperative topical streptomycin as a protective factor was effective in preventing postoperative surgical site infections in spinal tuberculosis (*p* < 0.05). Ahuja et al. [18] investigated the role of streptomycin powder in preventing surgical site infections in spinal tuberculosis and demonstrated that it was effective in The results proved to be effective in reducing the incidence of SSI. However, in our study, local irrigation of the lesion with streptomycin saline had no significant advantage for the prevention of SSI (*p* = 1). After our analysis, an important reason for this is the relatively small number of cases included in this study and the lack of representativeness of the overall distribution. On the other hand, the causes of SSI in spinal TB are multifactorial. In a cohort of 672 patients with surgically treated spinal tuberculosis [19], 54 developed SSI. multifactorial binary logistic regression analysis showed that irregular chemotherapy, incomplete lesion clearance, serum albumin concentration < 30 g/L, blood glucose ≥ 11.1 mmol/L and haemoglobin ≥ 20 mm/h were independent risk factors for SSI in STB patients. Therefore, future prospective randomized controlled trials in more centres and larger samples are needed to determine whether streptomycin rinsing of the lesion locally can prevent SSI.

Notably, our results show that localized streptomycin irrigation given to the lesion during surgical procedures for spinal tuberculosis effectively reduced the patient's postoperative ESR and CRP levels (both *p*-values < 0.05). This may be due to the deeper clearance of Mycobacterium tuberculosis, which allowed for a reduction in the inflammatory response of the organism. In general, ESR and CRP are positively correlated with increased inflammatory material in the body and their increase indirectly reflects an increased risk of postoperative infection. Streptomycin washout was able to reduce the mean duration of postoperative antibiotic use (*p* < 0.001) and length of hospital stay (*p* < 0.004) in patients compared to the control group. This is closely related to changes in inflammatory indicators.

In conclusion, to our knowledge, there are very few reports on the prevention of SSI in spinal tuberculosis with streptomycin. In clinical practice, antibiotics are usually administered orally or by intravenous drip, however, the concentration of the drug at the TB site is lower than in systemic intravenous blood, and the antibacterial effect is limited. The combination of previous studies and our results suggests that localized streptomycin irrigation or placement of streptomycin powder during spinal tuberculosis surgery can reduce the duration of postoperative antibiotic administration and the number of days the patient stays in hospital. This is important for increasing the effectiveness of spinal tuberculosis and reducing the emotional and financial burden on patients. However, the retention of streptomycin powder at the surgical site may have certain shortcomings: on the one hand, given the large amount of local blood and exudate that occurs after the lesion is cleared, it is very likely that the diluted streptomycin will be drained out of the body through the drainage tube, resulting in a decrease in the local drug concentration; on the other hand, the pharmacokinetics of local streptomycin at the surgical site has not been clearly studied. The dose of streptomycin powder is difficult to control, and if a large dose of streptomycin is given intraoperatively, it may have a potent and long-lasting local bactericidal effect. However, the concentration level in the systemic blood will be relatively increased, and this will most likely lead to some toxic adverse effects.

The solution of applying streptomycin saline flushing has the following advantages: (1) it can form a highly concentrated antibiotic environment in a small area with low blood concentration, avoiding the toxic side effects that may result from the retention of large doses of powder; (2) it can flush both local and superficial incisional tissues of deep lesions, covering a wide area and a large sterilization range; (3) the flushing will form a fluid flow with certain impact, and after the negative pressure suction head's The suction of the negative pressure suction head can remove residual pus and tissue debris in a timely and effective manner, effectively preventing the spread of contamination sources. Although there are problems with the short-lived efficacy of the streptomycin saline irrigation protocol, our study confirms that this method can effectively improve patient outcomes and is a safe and effective procedure for the removal of spinal tuberculosis lesions.

Deep postoperative spinal incisional infection is a more serious clinical postoperative complication of spinal surgery, and for many reasons, patients with spinal tuberculosis are more susceptible to this complication, which adds significant morbidity and financial burden to patients and adversely affects their mental status and quality of life. Although our findings show that the use of topical streptomycin irrigation of the lesion during surgical procedures for spinal tuberculosis is effective in controlling the level of postoperative infection, the average length of hospital stay and postoperative antibiotic use by patients. However, surgeons who want to better prevent the occurrence of SSI after spinal TB need to should adopt a variety of targeted interventions. This study was limited to a retrospective study with a small sample size and a short follow-up period, and higher level evidence-based studies are needed to inform clinical treatment planning.

## Data Availability

All data generated and analyzed during this study are available from the corresponding author on reasonable request.

## Abbreviations

SSI: Surgical site infection
ESR: Erythrocyte sedimentation rate
CRP: C-reactive protein
